# Development and evaluation of a wheelchair service provision training of trainers programme

**DOI:** 10.4102/ajod.v6i0.360

**Published:** 2017-09-08

**Authors:** Sara Munera, Mary Goldberg, Krithika Kandavel, Jonathan Pearlman

**Affiliations:** 1 El Comité de Rehabilitación, Medellin, Colombia; 2 Rehabilitation Science & Technology, University of Pittsburgh, United States; 3 Human Engineering Research Laboratories, Department of Veterans Affairs, United States; 4 Department of Rehabilitation Science and Technology, University of Pittsburgh, United States

## Abstract

**Background:**

In many countries, availability of basic training and continued professional development programmes in wheelchair services is limited. Therefore, many health professionals lack access to formal training opportunities and new approaches to improve wheelchair service provision. To address this need, the World Health Organization (WHO) developed the WHO Wheelchair Service Training of Trainers Programme (WSTPt), aiming to increase the number of trainers who are well prepared to deliver the WHO Wheelchair Service Training Packages. Despite these efforts, there was no recognised method to prepare trainers to facilitate these training programmes in a standardised manner.

**Objectives:**

To understand if the WSTPt is an effective mechanism to train aspiring wheelchair service provision trainers.

**Method:**

An action research study was conducted using a mixed-methods approach to data collection and analysis to integrate feedback from questionnaires and focus groups from three WHO WSTPt pilots.

**Results:**

Trainees were satisfied with the WHO WSTPt and the iterative process appears to have helped to improve each subsequent pilot and the final training package.

**Conclusion:**

The WHO WSTPt is an effective mechanism to train wheelchair service provision trainers. This programme has potential to increase the number of trainees and may increase the number of qualified service providers.

## Introduction

### Literature review

Of the world’s population, approximately 15% have a disability and 1% need a wheelchair (World Health Organization 2008), with a higher prevalence among people in developing countries (The World Bank 2015). Unfortunately, only 15% of those needing a wheelchair actually have one (World Health Organization 2011), even though literature demonstrates the importance of using wheelchairs to access the right of personal mobility and other human rights (Borg et al. 2012; May-Teerink 1999; Shore 2008). This issue will be more prevalent in coming years because of the ageing of the world’s population (Lee 2003) and the global increase in chronic health conditions (World Health Organization 2011). According to the United Nations Convention on the Rights of Persons with Disabilities (UNCRPD) (United Nations 2006), people with disabilities have the same rights, and should have equal opportunities like any other citizen. Wheelchairs and their related services are an important way to access the right of personal mobility. As stated by Borg et al. (2012), to support the implementation of the UNCRPD, research related to policies, service delivery, outcomes and international cooperation are needed.

Negative consequences can occur from not having an appropriate wheelchair, or receiving a wheelchair without the related recommended services. These consequences include the development of pressure sores, bad posture and low independence and self-esteem (World Health Organization 2012). This may also cause an impediment to education and employment because of the lack of appropriate assistive technology to enable social participation (McClure et al. 2009). According to the World Health Organization (WHO), significant barriers to the human right of personal mobility include discrimination against people with disabilities and a lack of trained wheelchair service personnel (World Health Organization 2008). To date, there is a gap in the literature related to how service provision occurs globally, but studies from various contexts recognise a need to improve it (Toro et al. 2012, 2016). To prevent these negative consequences, wheelchairs need to be delivered by people who are trained in an appropriate manner.

### Situational awareness: Too few people are trained in wheelchair service provision, resulting in poor-quality wheelchairs and services

In 2008, the WHO published the Guidelines for the Provision of Manual Wheelchairs in Less Resourced Settings (World Health Organization 2008). The aim of the document was to ‘promote personal mobility and enhance the quality of life of wheelchair users by assisting member states in developing a system of wheelchair provision’. The guidelines describe eight steps to facilitate appropriate wheelchair provision (referral, assessment, prescription, funding and ordering, product preparation, fitting and adjusting, user training and follow-up, maintenance and repairs) (World Health Organization 2008) and served as the foundation for the development of the Wheelchair Service Training Packages (WHO WSTP) (World Health Organization 2012, 2013). The WHO WSTP aims to improve wheelchair service provision, mainly in developing countries, to support the minimum skills and knowledge required by personnel involved in wheelchair service delivery at both the basic and intermediate level (World Health Organization 2012, 2013). The following training packages were published to meet this goal: WSTP-Basic in 2012, Intermediate in 2013 and managers and stakeholders in 2015.

Despite progress made by the WHO, limited coordination and training, as well as consistency in service provision efforts exist for less resourced settings. In these areas, research demonstrates challenges in wheelchair service delivery as well as dissatisfaction with wheelchair design and services (Visagie et al. 2015a, 2015b). A study conducted in South Africa revealed gaps between guiding documentation and service delivery in six out of the eight WHO steps. This suggests that in at least one area, despite having the WHO materials as reference material, because of contextual issues, service provision steps might not be adhered to consistently (Visagie et al. 2013). However, another study in Zimbabwe concluded that a comprehensive wheelchair service programme focused around training and proper service provision resulted in significant positive changes in user satisfaction with the wheelchair, and wheelchair services (Visagie et al. 2016). This suggests a wheelchair, when provided in an appropriate manner by a properly trained service provider, may help a person with a mobility impairment gain greater personal mobility and help fulfil other basic human rights.

Researchers have studied the importance of wheelchair training, mainly related to wheelchair skills (Best et al. 2005; Bonaparte et al. 2004; Coolen et al. 2004; Kirby et al. 2008, 2015) and wheelchair maintenance (Toro 2015). Training of trainers programmes have also been studied and proven to be effective in increasing competence and confidence in trainees (Smith et al. 2014). This model of training has proven to be cost-effective and sustainable in different settings and with multiple types of trainees, including service providers (Suhrheinrich 2014). To date, the efforts to train trainers in wheelchair service delivery have not been comprehensively described in the literature.

There are other entities delivering trainings in wheelchair service provision globally, including universities, non-governmental organisations (NGOs) and humanitarian organisations. Unfortunately, most of these organisations use different curricula and training methodologies without any standardisation (Free Wheelchair Mission 2016; LDS 2012; Motivation 2015, UCP Wheels for Humanity 2016). The same problem exists in the way wheelchair service providers and trainees’ knowledge is measured (Gartz et al. 2016). As a response to the lack of professionalisation and standards across the wheelchair sector, the International Society of Wheelchair Professionals (ISWP) was established. This organisation is working to increase awareness about wheelchairs and related services, establish product and service standards and coordinate training initiatives (International Society of Wheelchair Professionals 2017). ISWP recently developed and validated a Basic Wheelchair Service Provision Test as a method to measure competency (Gartz et al. 2016). ISWP is leading a follow-on effort to develop an Intermediate Wheelchair Service Provision Test and will be undergoing similar validation steps as conducted at the basic level.

### A possible solution: Development of a draft training of trainers programme and recognition process

Even with the advent of standardised wheelchair service training materials by the WHO and assessments by ISWP, to date there is no recognised method to prepare a cadre of trainers to facilitate the WHO WSTP trainings in a standardised manner and build capacity in service provision globally. Thus, the WHO WSTP WHO Wheelchair Service Training of Trainers Programme (WSTPt) was drafted as a next step in the WHO WSTP and ISWP professionalisation process, to build off of the foundation created by the basic and intermediate training programmes and assessments, respectively. The development of this training programme started in 2014 with a goal to increase the number of qualified trainers to facilitate the WHO WSTP (including basic, intermediate and managers and stakeholders) globally and encourage the proliferation of more qualified service providers.

The WHO WSTPt package, as well as the training process, was developed by experts using an iterative process that included material development by an expert panel and, later, a multi-stakeholder review to provide recommendations on prelearning materials and all session plans. Topics such as adult learning principles, diversity and cultural competency are included in the WHO WSTPt, as well as the use of a variety of audio visual tools to appeal to trainees with different learning styles. The WHO endorsement and publishing process ensures the training package was developed using a high-quality methodology and a transparent process (World Health Organization 2016) and allows the programme to be marketed on the WHO site, distributed to member states and partners and remain open-access. The training is designed to be held over a five-day period where the first two days focus on core knowledge to improve training skills. During the last three days, trainees have a practice delivery experience, in which they deliver, as lead trainers between two to four sessions, and serve as support trainers on three to five sessions for other trainees. Upon completion of the five-day session, the WHO WSTPt materials then encourage trainees to participate in co-training sessions with experienced trainers.

ISWP, similar to the assessments it developed to accompany the basic and intermediate levels, developed a comprehensive Trainee Competency Assessment (TCA) tool especially for this programme to assess the ability of new WSTP trainers against a set of eight preset competencies. The tool, encompassing a three-point scale and eight competencies, was developed in concert with the WHO WSTPt materials and validated by an expert panel of nine individuals from five different countries. The competencies include preparation, time management, delivery of WSTP materials, presenting, facilitating, giving or receiving feedback, managing group work and communication (where culturally appropriate). The TCA (see copy in Appendix 1) was designed to be used as the assessment for the ISWP trainer recognition process, as WHO as an entity does not certify, recognise or assess individual competency in wheelchair service provision. The TCA is used by trainers in both the training and co-training to assess a trainee’s delivery skills. The trainer gives the trainee the completed TCA at the end of both the training and co-training.

**FIGURE 1:**
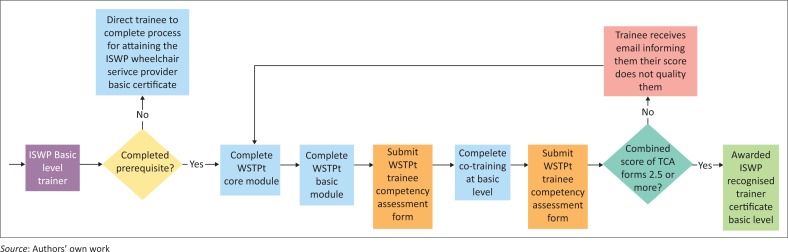
ISWP training recognition pathway.

The ISWP trainer recognition process was also developed by the expert panel and as an example, the ISWP Basic Level Trainer pathway is demonstrated in the flow chart as shown in Figure 1. Similar paths exist for both the intermediate and managers and stakeholders levels. If a trainee receives an average score of 2.5 or higher on the TCA as rated by his or her trainer from both the WSTPt and the co-training (may be the same or different people), they are recommended to advance through the process and receive ISWP trainer recognition status, respectively.

While the draft WHO WSTPt and ISWP Trainer Recognition Process both serve as possible avenues to increase the number of trainers worldwide, prior to this study, neither had been evaluated. Therefore, an action research study, with one primary research question, was initiated by a team of colleagues to assess and potentially improve upon the WHO WSTPt and ISWP Trainer Recognition Process.

Research question: Is the *WHO WSTPt*, including the *WHO WSTPt materials*, an effective mechanism *to train* aspiring wheelchair service provision trainers?

## Research methods and design

As defined by Reason and Bradbury (2001):

‘action research seeks to bring together action and reflection, theory and practice, in participation with others, in the pursuit of practical solutions to issues of pressing concern to people, and more generally the flourishing of individual persons and their communities.’ (p. 3)

Action research can be particularly effective in the evaluation of training programmes and has been well documented in the literature where an iterative process with continuous feedback from trainers and trainees can improve training initiatives based on experience (Lecompte & Preissle 1993; Milano & Ullius 1998). Mezirow (1991) identifies three forms of reflection: content, process and premise, where content reflection is the substance; process reflection is the strategies, procedures and how things are being done; premise reflection is the underlying assumptions and perspectives (Mezirow 1991). Therefore, an action research study was designed using a mixed-methods approach to data collection and analysis to integrate feedback from questionnaires (surveys and forms) and focus groups to evaluate the WSTPt Programme *content*, WSTPt and ISWP trainer recognition *processes* and overall *premise* to determine stakeholders’ perspectives.

In order to develop this action research project, the WHO WSTPt was facilitated across three pilot sites using the WHO WSTPt materials and ISWP Training Recognition Process (including the TCA). The pilot sites were selected based on strong in-country partner organisations and access to participants with satisfactory English language proficiency. Likewise, an effort was made to select culturally diverse and different socioeconomic settings to test the generalisability of the training and assessment methodology. Thus, Nairobi, Kenya (lower-middle income), Cape Town, South Africa (upper-middle income), and Bangkok, Thailand (upper-middle income), were identified as meeting all criteria and were selected for the three pilot sites to be conducted in 2016. In each of the three pilots, trainees were recruited from rural, peri-urban and urban settings. Additionally, while the same materials were used for the core in each pilot, different modules of the WSTPt (basic, intermediate and managers and stakeholders) were facilitated to further test generalisability of the method itself. For pilot 1 in Kenya, the options were WSTPt basic and managers and stakeholders, for pilot 2 in South Africa, WSTPt intermediate and managers and stakeholders were held, and for pilot 3 in Thailand, WSTPt basic and intermediate were facilitated.

Up to 25 trainee subjects were recruited for each pilot. Trainees’ inclusion criteria were (1) satisfactory English language proficiency, (2) currently working in the area of wheelchair service provision and (3) passed the ISWP Basic Wheelchair Service Provision Test (Gartz et al. 2016). Secondary criteria included residency in or ability to train in the region where the pilot was held. The research team was not involved in running the training or selecting the trainees to help ensure independent and objective feedback.

As described in the abstract background section, the pilot training followed the WSTPt methodology of five days of training including a mix of didactic training on adult learning theory and wheelchair service provision and practice delivery sessions. Following best practice in action research (French 1999; Lewin 1946; Reason 2001), an iterative approach that solicited feedback from trainers, trainees and observers was employed to improve the methodology and material, so that feedback could be reflected upon by the joint team and used to immediately improve the subsequent training. The following data collection methods were employed: trainee satisfaction surveys, TCA forms (completed by trainers during each of the trainees’ practice delivery sessions) and focus groups of both trainees and trainers. Table 1 describes each data collection tool or method.

**TABLE 1:** Data collection methods.

Data collection method	Description
Trainee satisfaction surveys	Surveys were delivered to trainees at the end of the WHO WSTPt. The satisfaction survey used a Likert scale from 1 to 5 (1 = poor, 2 = fair, 3 = good, 4 = very good, 5 = excellent) to evaluate the overall programme content, PowerPoint slides, trainee handbook, group activities, facilitation of activities and preparation material received. Additionally, there was a self-assessment question asking the confidence in delivering the WHO WSTP before and after the training programme.
Focus groups	At the end of each training day, focus groups were held with trainees. On the last training day, additional focus groups were held with trainees and trainers separately. A professional coach or observer from the WHO WSTPt conducted the sessions. During the 45 min focus groups, trainees gave feedback related to the training materials and methodologies; this information was documented and later coded by ISWP.
TCA forms	Trainers assessed trainees on different dimensions to provide feedback on how to improve training skills. Trainees received a final grade allowing them to advance to the next step in becoming a trainer. These forms were reviewed for completeness, overall scores were averaged and comments were coded by ISWP. To date, ISWP has received few TCAs, these results will be reserved for a future manuscript.

*Source*: Authors’ own work

## Results

As described above, the pilot trainings were held in Nairobi, Kenya, Cape Town, South Africa, and Bangkok, Thailand, and had an average of 5 trainers, 22 trainees and 6 observers to participate in each training. Table 2 shows the number of attendees at each training.

**TABLE 2:** Roles and number of attendees for each training.

Pilot	Location	Roles	Number of attendees (female, male)
1	Nairobi	Trainees	20 (11, 9)
		Observers	5
		Trainers	5
2	Cape Town	Trainees	23 (8, 15)
		Observers	6
		Trainers	6
3	Bangkok	Trainees	22 (11, 11)
		Observers	6
		Trainers	4

*Source*: Authors’ own work

The role of the trainers, who were all advanced-level wheelchair service providers and experienced WHO WSTP trainers, was to deliver the content outlined in the WSTPt training package according to the guidelines. The observers, all experienced evaluators from ISWP and other NGOs in the wheelchair sector, were assigned to take notes during the sessions and evaluate what could be improved for next trainings in terms of content, time and methodology. The observers from ISWP were also responsible for collecting each of the metrics including trainee satisfaction surveys and TCA forms. Data from observers and the TCA will be included in a future manuscript.

**FIGURE 2:**
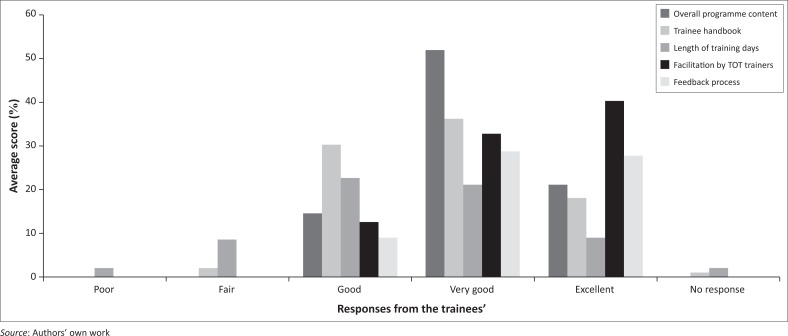
Trainees’ average satisfaction ratings across Pilot 1 (Nairobi), Pilot 2 (Cape Town) and Pilot 3 (Bangkok).

Figure 2 presents select satisfaction survey results from the three pilots and suggests that trainees were satisfied with the overall programme content, trainee handbook, facilitation by trainers and feedback process. In general, most of the responses were clustered around ‘Good’ ‘Very Good’ and ‘Excellent’. There was some discrepancy with trainees’ perception of the length of the training day, with only 30% suggesting it was ‘Very Good’ or ‘Excellent’. The ‘overall programme content’ item was consistently rated ‘Very Good’ on average across all the three pilots. The ‘facilitation by WSTPt trainers’ item was also consistently rated ‘Excellent’ across all the three pilots.

According to the self-assessment question (‘How confident do you feel to deliver the WHO WSTP?’) that was asked on the trainee satisfaction surveys, there was an increase in confidence to deliver the training after the WHO WSTPt (as shown in Table 3). The percentage increase represents the difference between the mean scores before and after the training. Participants reported an average of 80.6% confidence to deliver the WHO WSTPt after the training.

**TABLE 3:** Self-perceived confidence to deliver the WHO WSTPt.

Pilot	Location	Type of training	Average percentage (%) increase in confidence across trainees
1	Nairobi	Basic	71
		Managers and stakeholders	35
2	Cape Town	Intermediate	67
		Managers and stakeholders	154
3	Bangkok	Basic	76
		Intermediate	81

*Source*: Authors’ own work

Qualitative feedback was collected from trainees from both trainee satisfaction surveys and focus groups after every WHO WSTPt session. For the satisfaction survey data, one ISWP researcher who also served as a WSTPt observer analysed and grouped comments into five themes through a first and second cycle coding method based on frequency of occurrence (Saldaña 2015): knowledge, resources, feedback process, trainers and significance of the training. Table 4 includes some of the most frequent comments from trainees in different pilot locations.

**TABLE 4:** Frequent themes from trainees from satisfaction surveys.

Theme	Comments or general description	Illustrative quote	Training location
Knowledge	WSTPt helps trainees identify errors in their own practice. WSTPt conveys the critical information needed for training independently.	‘For us we have been training and we think we are good but then we learn we’ve been making mistakes and we know we need to work on this or this.’ ‘The programme content is good and it prepares well for the practice and training modules.’	Nairobi and Cape Town
Resources	Resources (slides and trainee handbook) were helpful for preparing training sessions.	‘They have the content which was quite educative and helpful in understanding the programme.’ ‘Provided essential guidelines.’ ‘Great cues for the trainer and guidance for each slide and methods of presentation.’	Nairobi, Cape Town, and Bangkok
Feedback	Feedback was provided in a respectful manner from other trainees and trainers and improved trainees’ skills. Trainees also valued helping others better their skills.	‘Makes it possible to become better and also help others better their skills.’ ‘The one-on-one feedback provided more insight towards improvement.’ ‘Good collaboration of team and observers as well as the trainers for the participants to improve better and learn more skills to be competent.’	Nairobi, Cape Town, and Bangkok
Trainers	The trainers were of high quality and clarified complex content. In general, experienced trainers received higher praise and more complimentary feedback than inexperienced trainers.	‘They took time to clarify aspects which helped me to understand the content.’ ‘Trainers gave a good example and role modelling.’ ‘Good in guiding and help a lot.’ ‘Every trainee was able to know his strength and weaknesses.’	Nairobi, Cape Town, and Bangkok
Significance	Trainees independently identified the significance of participating in the training to their future work.	‘In our own country, we have a big population and most people are distributors of the chairs they don’t know. So, this is very helpful for us to make a plan for awareness.’	Nairobi

*Source*: Authors’ own work

The other qualitative data were obtained from trainee focus groups conducted by a professional coach or observer from the WHO WSTPt stakeholder author group. While some programme modifications were derived from trainee feedback from the satisfaction surveys, the majority of modifications were made based on trainees’ focus group feedback, as these sessions focused specifically on what could be done to improve the programme. Recommendations and changes to improve the programme were collected through focus group sessions at the end of each training day, as well as summative focus groups on the last training day. Notes were taken by both an ISWP researcher and the other programme observers and provided to the lead trainers. This allowed real-time changes to the programme content to occur both within a particular training as well as more systematic changes for the next pilot. A summary of these recommendations and modifications is shown in Tables 5 and 6.

**TABLE 5:** Modifications made during the second pilot based on the first pilot feedback.

Recommendation from Pilot 1	Modifications made on Pilot 2
Improve timing of practice sessions	More guidance was delivered to trainees on how much time needs to be dedicated to each session
Communicate early about the sessions trainees will lead, so trainees have ample time for preparation	During the first day, trainees were randomly assigned specific content
Improve diagrams and explanation of the ISWP recognition process so that trainees are clear and trainers have the information they need to respond to all of the TCA items	ISWP training staff provided content
Provide fact sheets on what ISWP is about and invite trainees to become members	ISWP training staff provided content
Modify some of the session content, especially the sessions on ‘adult learning principles’ and ‘cultural competency’	Adult learning principles: An activity was added to relate the learning principles to the WHO WSTP materials Cultural competency: Examples about different contexts were provided
Provide more guidance to promote collaboration between lead trainers and co-trainers in the practice delivery sessions	Added content about collaborative practice into one of the sessions Trainers provided more mentoring during the practice delivery sessions

*Source*: Authors’ own work

**TABLE 6:** Modifications made during the third pilot based on the second pilot feedback.

Recommendation from Pilot 2	Modifications made on Pilot 3
Allocate more time for trainees to prepare the sessions they need to present	At the end of the first two days, 1 h for preparation was allocated where trainees can interact with the trainers.
Inform trainees about the sessions they will deliver with more time for preparation	Trainees were informed about their sessions on the first day instead of informing one day before delivering.
Include more description of the competencies in the TCA	A description of each of the competency domains was added.
Modify the TCA rating system to be 0–5 instead of 0–100	The rating scale was changed from 0 to 5 and average threshold was updated to 2.5/5 instead of 60/100.

*Source*: Authors’ own work

## Trustworthiness

The results of this study were based on an independent evaluation of the WHO WSTPt and the ISWP Trainer Recognition Process. While the evaluators were collaborators of the stakeholder author group, they were not involved in the design of the WHO WSTPt materials or process. The evaluators provided feedback into the ISWP Trainer Recognition Process and TCA, but were not involved in completing any TCAs (the trainers completed these on behalf of the trainees). It is possible that because of the evaluators’ involvement in the ISWP Trainer Recognition Process and TCA, trainees may have felt slightly inhibited in sharing candid feedback through both surveys and focus groups.

The reliability of the qualitative data from the satisfaction surveys was strengthened through first and second cycle coding by a single researcher, where themes were later verified by looking at separate passages of data. Where new themes emerged, they were added, and then the same process was repeated. A second researcher reviewed the themes and illustrative examples for consistency and repeated the same process.

A participant–observation ethnographic approach was taken to conducting and observing the focus groups. The ISWP researchers who were involved in the focus group were considered to be a part of the community and the facilitators (professional coach or observers) had a generally strong rapport with the trainees as well to support a community of sharing. In this way, we believe that the trainees were open to sharing candid feedback because of the opportunity they had to improve the programme experience for future trainees and the betterment of the wheelchair sector as a whole. The trainees’ illustrative passage in Table 4 provides evidence of this belief.

The TCA was reviewed for content validity by both the evaluators and stakeholder author group (authors and editors of the WHO-WSTPt material) and underwent several revisions. Prior to this study, the TCA has not been reviewed for intra- or interrater reliability; however, once the content and scoring is updated based on this action research study, both intra- and interrater reliability will be conducted.

## Discussion

In response to our research question, there is ample quantitative and qualitative evidence to suggest that the WHO WSTPt and its materials are an effective mechanism to train aspiring wheelchair service provision trainers in terms of both *process* and *content*. This is demonstrated through gains in trainee knowledge, skill and confidence.

First, the WHO WSTPt seems to have increased trainees’ wheelchair services training knowledge and skills. As suggested by a trainee from Nairobi:

‘The whole process helped. Watching the trainers and […] other colleagues, trying to apply these […] on your training.’

Therefore, trainees seemed to value the *process* with the opportunity to first learn the content and later practise skills by modelling trainer behaviour. In line with research on how to improve wheelchair services, even experienced trainees gained new knowledge and skills through the training (Visagie et al. 2015a, 2015b; World Health Organization & Imperial College London 2015). For example, one trainee cited:

‘We have been training and we think we are good but then we learn we’ve been making mistakes and we know we need to work on this or this.’

This is an important finding, suggesting that the WSTPt can be used as both initial training and continued professional development for experienced trainers as research suggests that the quality of teaching depends on teachers continuing to learn as teaching contexts, student behaviour and expectations of teachers change (Day 1999). In other words, the range of wheelchair service provision contexts, knowledge and skill wheelchair service providers possess and expectations thereof that are placed on trainers will only continue to expand in coming years. Likewise, trainees’ feedback also suggested that the WHO WSTPt has adequate *content* to accomplish its goal of improving knowledge. For example,

‘It is a very informative package and learnt a lot from the programme.’

The exercise of providing feedback seemed to be a particularly helpful component and part of the *process* as it allowed trainees to recognise their strengths and areas of improvement, as suggested by trainees:

‘Every trainee was able to know his strength and weaknesses.’

Feedback makes it possible to become better and also help others better their skills.

Peer feedback was found useful, but according to trainees:

‘One-on-one feedback provided more insight towards improvement.’

Second, the WHO WSTPt seems to have increased trainees’ confidence to deliver the WHO WSTP. For example, one trainee cited about the whole *process* that:

‘The programme is good and it prepares well.’

Training confidence could be especially important when it comes to co-training in trainees’ home countries. The replication of trainings in different settings could increase awareness about the importance of wheelchairs and wheelchair services to improve social participation, as well as the steps to ensure adequate wheelchair services. For example, one trainee cited:

‘In our own country we have a big population and most people are distributors of the chairs they don’t know. So this is very helpful for us to make a plan for awareness.’

While the training *process* was helpful as described above, the training *content* also seemed to be helpful for trainees to learn how to deliver trainings. The trainee handbook was particularly valuable according to trainees in Nairobi:

‘It was perfect – it’s the best part of the training resources.’

It was an important resource, according to trainees as it

‘points out key learning/preparation points and all corrections for errors in the manual.’

Trainees in Cape Town reiterated that the handbook

‘provided essential guidelines which to me effectively helped in understanding better the programme.’

While results were somewhat uniform across the pilots and supported the effectiveness of the WHO WSTPt, more positive comments were received from the trainings with trainers who had more experience, as suggested by these sample comments:

‘Trainers have been excellent in their presentations.’

‘They took time to clarify aspects which helped me to understand the content.’

‘Trainers gave a good example and role modelling.’

Additionally, experienced trainers allowed for more peer interaction and social construction of knowledge as suggested by this trainee:

‘They also allowed room for shared experiences, they were not uptight and available for consultation and feedback.’

This may suggest a need for a recommendation from ISWP on who is prime to lead WHO WSTPt based on their experience, and how aspiring WHO WSTPt trainers can gain skill and experience to enable them to best prepare trainees.

In summary, the WHO WSTPt appears to support emerging trainers’ knowledge, skill and confidence to deliver trainings in support of an affirmative answer to our research question, which investigated the overall programme effectiveness. The iterative action research approach to this study may have resulted in improved satisfaction scores and more complimentary trainee comments with each WHO WSTPt iteration, suggesting the intervention improved over time and as a result of the feedback received. These findings relate to previous research suggesting the importance of an iterative approach to developing training programmes (Milano and Ullius 1889). This research can help propel the wheelchair sector forward to encourage more and better prepared trainers to support trainings in more regions and improve wheelchair service delivery in order to support the implementation of the UNCRPD (Borg, Lindström & Larsson 2011).

## Limitations of the study

This action research study resulted in several practical improvements to the WHO WSTPt but has several limitations including how data were collected, by whom the data were collected and generalisability of data. First, because of the incremental nature of the programme and evaluation approaches, data were not collected in a uniform way in terms of both data collection tools and subject groups, which may bias the interpretation of changes implemented between pilots. The data collection tools, along with the training content itself, were iteratively modified between pilots, making direct comparisons challenging. While trainee satisfaction surveys did not include names, one sheet was collected per trainee to ensure that feedback was obtained from each trainee (one sheet per trainee). Because of this, trainees may have been compelled to respond in a more favourable manner, knowing that while anonymity was encouraged, it may not have been guaranteed.

Second, the author group (authors and editors of the WHO WSTPt material) observed sessions they authored and occasionally trained on sessions they did not author, which may have biased their interpretation of participant learning outcomes, feedback provided during focus groups and the way the trainees learned the material. This may also threaten generalisability of the approach as the author group has an informed perspective that another trainer in the field may not. Similarly, both author group members and trainers from the other pilots served as observers, either at previous or subsequent trainings. Therefore, they also had an informed perspective related to the type of feedback provided based on their prior experiences and knowledge of the package, as well as potential undue influence of the performance of both trainers and trainees. The trainer’s teaching style and performance were not specifically monitored or assessed which could have also limited how and what the trainees learned.

Third, because the training and evaluation was conducted on a relatively small sample, in only three geographic locations, and solely in English, the results may not be generalisable beyond the settings in which the study was conducted. Thus, the assumption of modifications leading to improvements is a limitation to this study, as some changes that were suggested by stakeholders may be limited to a particular region or not universally valuable.

After finishing the WHO WSTPt training, and as a formal part of the process, trainees are recommended to co-train with a more experienced trainer on the package they are learning to train (basic, intermediate, managers or stakeholders). This study only focused on evaluating the WHO WSTPt training component, in part because of funding restrictions which has limited the opportunity to support trainees in co-training experiences. Therefore, an additional limitation is that the results solely reflect a component of the WHO WSTPt, and not the full programme. Similarly, if co-trainings continue to not be available, trainees will struggle to complete the full programme as intended, limiting its utility to the practical goal of proliferating trainers prepared to facilitate the WHO WSTPt.

## Conclusion

The iterative action research approach used to evaluate the WHO WSTPt demonstrated an improvement of the training programme *content* and *process* from one pilot to the next. The WHO WSTPt appears to be successful in increasing trainees’ knowledge, skills and confidence to deliver trainings prior to the trainees’ co-training experience. This standardised training package, by increasing the number of trainers conducting WHO WSTP worldwide, may assist in training wheelchair service providers uniformly and appropriately to ensure that wheelchair users are provided with high-quality service and products.

## Future work

Future work will include how to improve the content of the WHO WSTPt, including exploring other training of trainers materials to identify different ways to improve engagement and peer interaction, relevance and value of ‘modelling’ good trainer techniques, as well as knowledge sharing tools and methods. Feedback from participants or trainers of other training of trainers programmes in healthcare may also provide helpful feedback, because they would have a basis of comparison that would not be possible for our population who is unlikely to have completed another training of trainer programme in any healthcare field.

The impact of the WSTPt varied between experienced and inexperienced trainees but this finding was not further explored in this study. More research is needed to better understand this difference and promote better skills for those who are less experienced. Mentoring may serve as a mechanism to fill this gap and should be further explored.

Additionally, we plan to further explore trainee outcomes. To date, 17 trainees have completed co-trainings, and at least 10 trainees have co-trainings planned by June 2017. This is a disparity in the WHO WSTPt, as suggested above that a co-training is a required component. Additional data will be collected on this process and TCA forms will be analysed. Future analyses may compare trainee performance on TCAs across pilots, future trainings, different geographic areas and whether differences exist based on important participant characteristics such as education and experience level, English language proficiency, the type of organisation they represent and whether learning outcomes vary based on particular trainee learning style. To make the WHO WSTPt accessible to a broader set of wheelchair service providers, additional languages and validation processes are needed.

Research is recommended to monitor the overall implementation of the training and measuring the effect it has on trainers, service providers and ultimately wheelchair users. This may include how trainees have used the WHO WSTPt skills and research to identify if the programme has significance in increasing the number of trainings that are being held, the quality of those trainings, how training impacted trainees long-term and ultimately the wheelchair delivery process.
